# Supplementary figures and images for: Rapid adaptive evolution of avian leukosis virus subgroup J in response to biotechnologically induced host resistance
Source: PLoS Pathog. 2024 Aug 15;20(8):e1012468. doi: 10.1371/journal.ppat.1012468 (PMC11349186; doi:10.1371/journal.ppat.1012468)

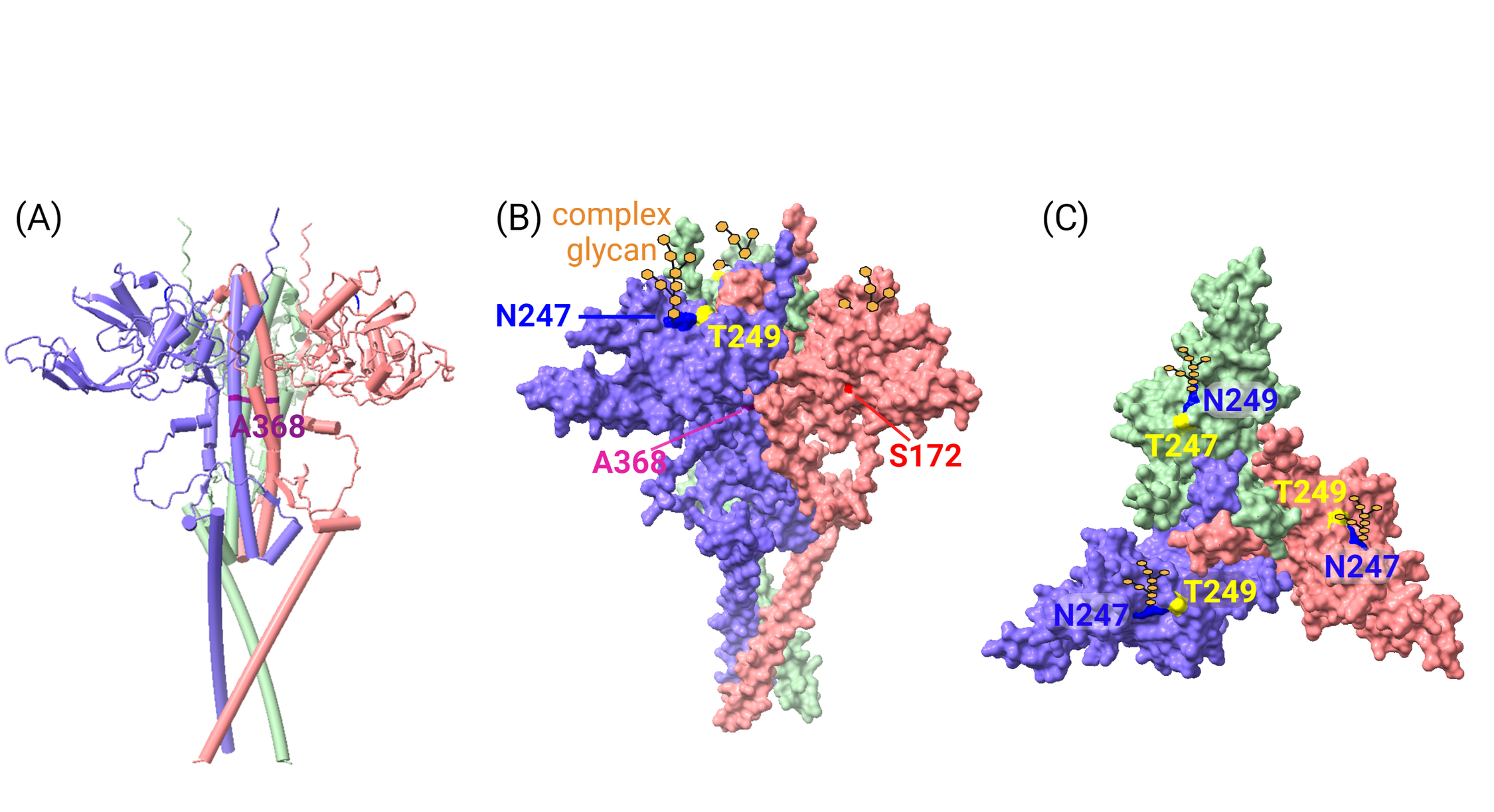

Supplement: S1 Fig — Each monomer is marked with a different color. Envelope is displayed from the side view as (A) a cartoon with cylinders and stubs, (B) with apparent molecular surface, and (C) from the top view with apparent molecular surface. Figure was created with Biorender.com. (TIF) [file ppat.1012468.s005.tif]

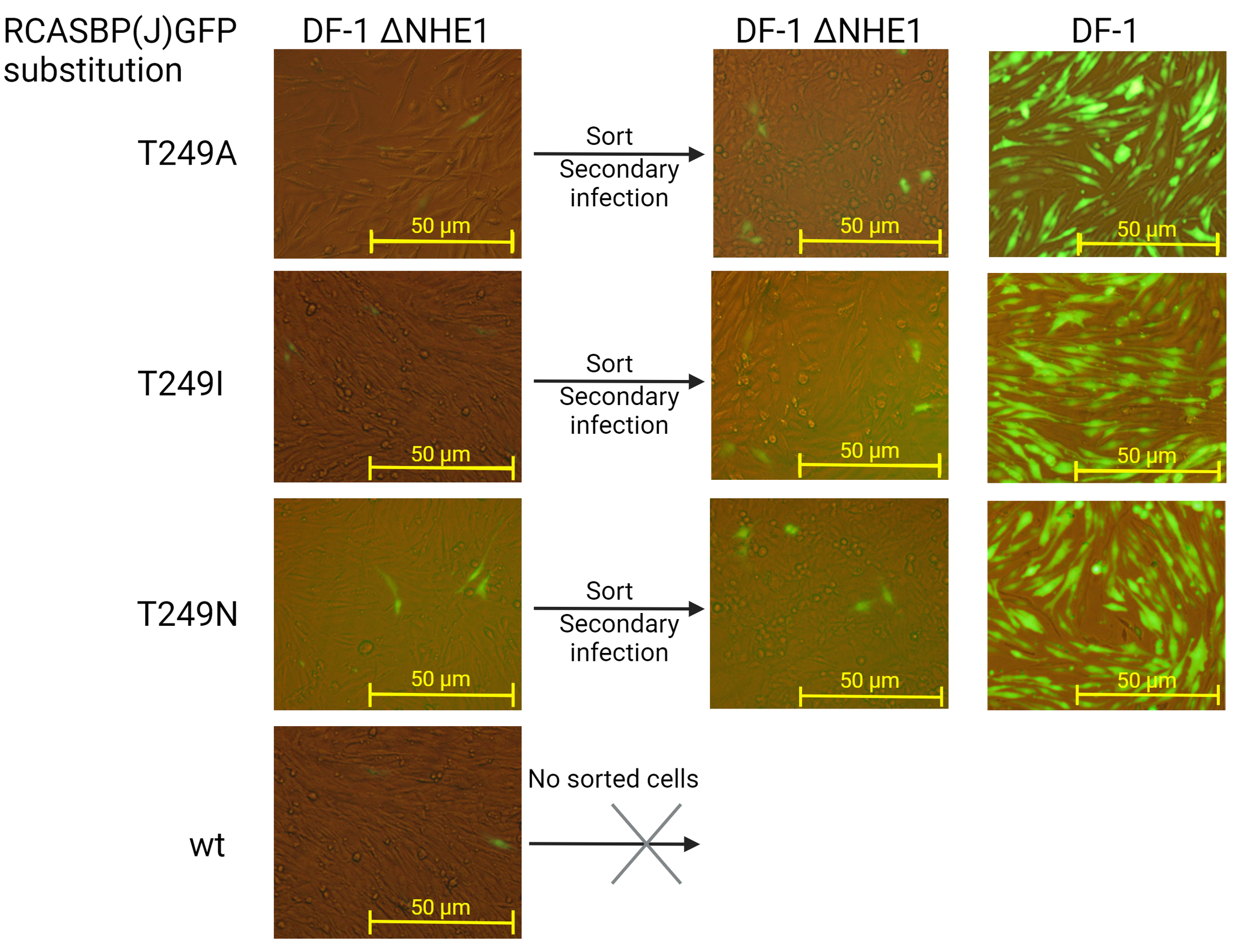

Supplement: S2 Fig — Individual GFP-positive DF-1 ΔNHE1 cells infected with adapted RCASBP(J)GFP variants were sorted. Collected virus stocks were used for secondary infection of DF-1 ΔNHE1 and DF-1. Representative photos of infection with RCASBP(J)GFP variants carrying the T313A, T313I, or T313N substitution are shown. Infection with other adapted virus variants carrying substitutions S236L, N311S, N311D, and N313T and with other cells (DF-1 Δ22 and DF-1 Δ4) were performed as well. Figure was created with Biorender.com. (TIF) [file ppat.1012468.s006.tif]

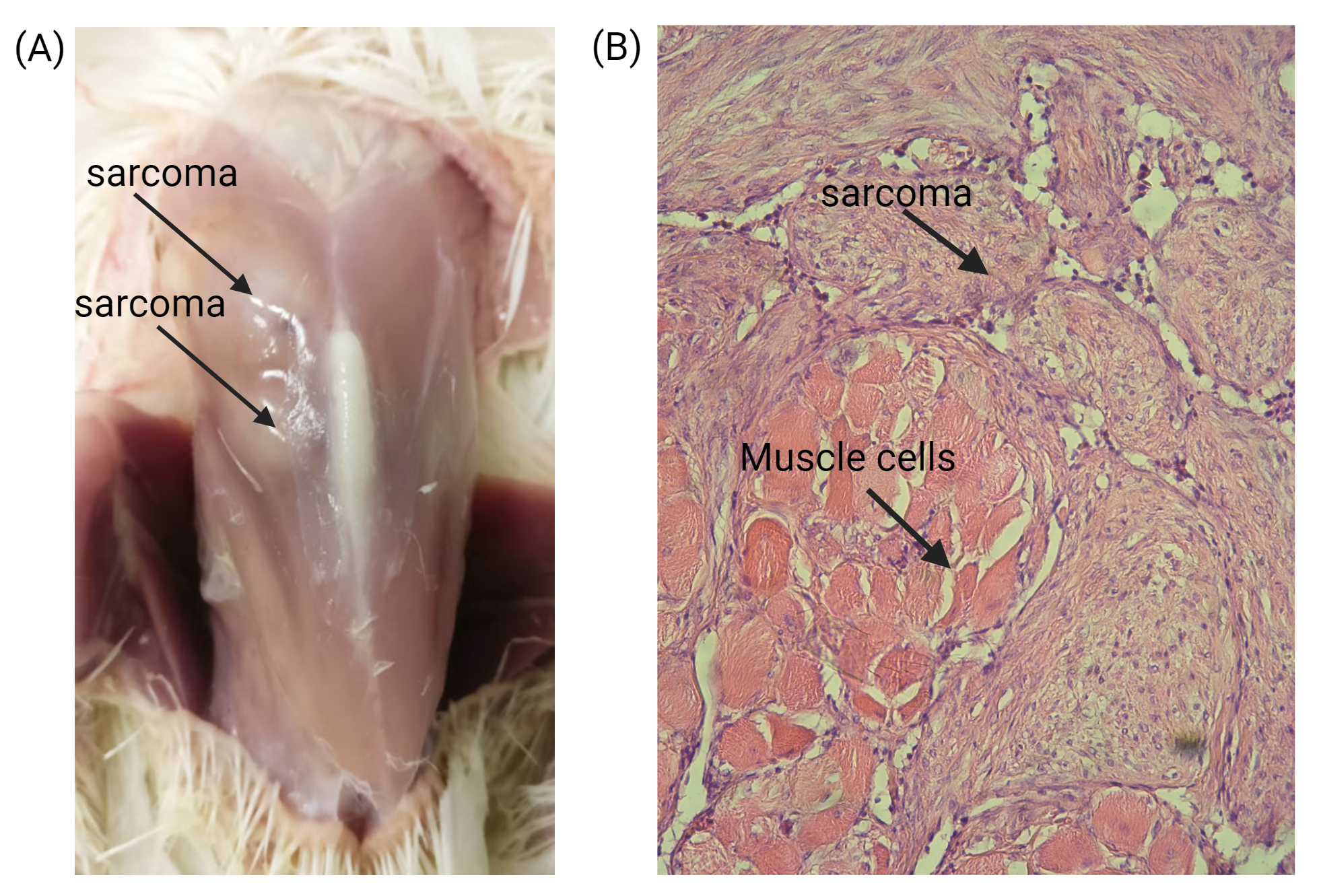

Supplement: S3 Fig — (A) Sarcoma in situ in the left pectoral muscle and (B) haematoxylin and eosin staining of a tumor section. Figure was created with Biorender.com. (TIF) [file ppat.1012468.s007.tif]
