# Supplementary material for: Rapid adaptive evolution of avian leukosis virus subgroup J in response to biotechnologically induced host resistance
Source: PLoS Pathog. 2024 Aug 15;20(8):e1012468. doi: 10.1371/journal.ppat.1012468 (PMC11349186; doi:10.1371/journal.ppat.1012468)
Supplement: Attachment — Submitted filename: response to reviewers.docx [file ppat.1012468.s009.docx]

Prague, July 22, 2024

Dear editors,

Please find below our responses to the comments and objections raised by reviewers. We have done our best to improve our manuscript according to their comments, and we hope you find our responses sufficient. We greatly appreciate the insightful and positive feedback from all reviewers.

Jiri Hejnar

(on behalf of the authors´ team)

# Part I - summary:

## Reviewer #3:

1. The only request I have to improve the readability of this manuscript is that the basic systems should be better explained up front. It is quite hard work to understand exactly what the viruses/ reporter systems used here are and what the assays are too. Figure 1 is a welcome schematic, but what IS the RCASPB GFP vector? To readers who are not familiar with these surrogate systems this needs explaining in the text at the start of the result section.

To improve the understanding of our manuscript we have added an explanatory sentence on RCASBP(GFP)J vector as recommended to the result section and reference to Fig. 2A with a vector scheme (line 103 - 104)

RCASBP(J)GFP is an ALV-based replication competent vector of subgroup specificity J that transduces GFP (Fig. 2A).

2. Similarly the complementation assay presented in figure 2F is very nice evidence that the mutated envs do not use alternative receptor but still rely on NHE-1. This assay is very important because the ALV family switch receptors during diversifying evolution. However the text describing this assay is minimal and the reader has to work hard to understand that a wild type reporter expressing ds red has been generated and used in coinfection studies, please embellish the text within the results section to explain so the reader does not have to interrupt the flow but cross referencing back and forth into methods section.

We have added an explanation of the complementation/interference assay into the result section (lines 187 - 196).

…we have performed the interference assay based on competition for receptor and entry interference between viruses of the same subgroup. We preinfected DF-1 cells either with all adapted variants of RCASBP(J)GFP and, two weeks later, we challenged the preinfected cells with wt vector carrying a dsRed fluorescent marker (RCASBP(J)dsRED). We also performed the reciprocal assay with wt RCASBP(J)dsRed as preinfection virus and adapted variants of RCASBP(J)GFP as a challenge. FACS analysis showed no evidence of GFP/dsRED double positive cells in the superinfected DF-1 culture. This strong interference suggests that the adapted variants use the same receptor NHE1 (Fig. 2F). As a control, we used the B subgroup RCASBP(B)GFP vector, which did not interfere with any of the J subgroup viruses.

3. Alphafold is used to show the position of the mutations in emergent viruses supp figure S1. Residues 311 and 313 are clearly poised to interact directly with the NHE-1 receptor. However, some other mutations are not- this suggests that increased protein expression transport or stability might also confer some way to infect cells with the suboptimal receptor- did the authors check for protein expression of the mutated envs?

Neither protein expression nor protein stability was tested. However, given the same efficiency of viral replication in the DF-1 cell line, adaptive mutations do not appear to significantly affect these properties *in vitro*. The main difference between DF-1 cells and W38^-/-^ fibroblasts is in the receptor, so it is unlikely that Env-J stability or export would differ between these two cell lines. See also the answer to major point 1 of the reviewer No. 1.

We have added a paragraph on this topic to the Result and Discussion sections.

Results (lines 176 - 178):

S236L, T313A, T313I, and A432T are substitutions that change the hydrophobicity of the side chain, while N311D and N311K are substitutions that change the charge of the side chain.

Discussion (lines 357 - 363):

In contrast, the other two mutated residues are not protruding and probably are not involved directly in the receptor binding. Both S236L and A432T represent substitutions that change the hydrophobicity of the side chain, which could impact the protein folding and influence other steps of virus entry into the cell, such as the membrane fusion process. Mutations can also affect virus stability or protein export efficiency, but given the same viral replication efficiency in the DF-1 cell line, the mutations do not appear to have a significant effect on these properties *in vitro*.

4. In figure 3 chickens are infected with the virus and tumours are induced even by wild type virus in W38-/- chickens Did the authors sequence virus in these tumours after day 30? Additionally did the authors sequence virus in chickens infected with other mutants in which high numbers of tumours were present? Perhaps further mutations have appeared?

As described in Methods, sarcomas in chickens were induced by a defective acutely transforming virus rescued from QT6 cells by a replication-competent helper, which adds the J-specific envelope glycoprotein. DNA from selected tumors was isolated and screened for the presence of RCASBP(J)GFP proviruses. Only the v-src-transducing replication-defective BH-RSV virus was detected in the tumor DNA, with no RCASBP(J)GFP sequences found. Technically, we detected the pol-src sequence from BH-RSV in all tumors. We however did not detect any env-GFP sequences in tumors (see Fig. 1 added to this letter). This confirmed the assumed mechanism and suggested that the replication-competent helper virus has been gradually eliminated. Elimination of replicating virus is not surprising and can occur by the same mechanisms as the clearance of primary viremia in an immunocompetent host (chicks at a few weeks of age). It was therefore not possible to sequence this virus. After all, following the further evolution of the virus was not the purpose of this study. Therefore, we provide the virus detection in tumors just as an explanatory figure in this letter.

**Fig 1.: Detection of BH-RSV and RCASBP(J)GFP in chicken tumors by PCR.** Each number represents one infected chicken. CB and CC are chicken inbred lines used here as negative controls. Primordial germ cells of CB origin were used for W38^-/-^ chicken preparation and have thus a very similar genetic background including the content of endogenous retroviral loci.

In future experiments, it would be interesting to prepare an RCASBP(J)src vector instead of using the pseudotyped virus. Such a system would allow us to track the occurrence of additional mutations. The current study shows that at least two single nucleotide mutations are sufficient to overcome the W38^-/-^ chicken resistance *in vivo*.

A single tumor induced by a wt virus in W38 -/- chicken remains an unexplained exception. We have never observed an analogous situation in our previous experiments [(Koslová et al. 2020, 2021)](https://paperpile.com/c/PDkPur/Udo4Z+YfPV). We have added a paragraph into the discussion where we speculate about a receptorless cell entry and the hypothetical rescue of v-*src* transducing virus by an endogenous retrovirus of different subgroup specificity.

The following paragraph was added into the Discussion (lines 379 - 386).

Surprisingly, in one instance, a tumor was induced in a W38^-/-^ chicken by the wt variant of the pseudotyped virus. This occurrence was an exception that did not appear in the previous study nor recurred in subsequent experiments. Unfortunately, due to the use of a pseudotyped virus, the explanation of the tumor induction is only hypothetical. We assume receptorless cell entry of the transforming virus and/or rescue of v-*src* transducing virus by an endogenous retrovirus of different subgroup specificity. Receptorless entry is also indicated by our *in vitro* experiments. (Fig 4B, Fig S2). Using high-multiplicity infection, the receptorless virus entry was sporadically observed in vivo as well [(Elleder et al. 2004; Lounková et al. 2017)](https://paperpile.com/c/PDkPur/0WGc+Ti8q).

The likelihood of mutation within the RCASBP(J)GFP vector was minimal due to its transfection into 16Q cells, known to be resistant to the RCASBP(J)GFP virus. Consequently, virus replication and mutation could not occur during Env-J pseudotype virus production. Additionally, the W38-/- chicken, resistant to the wt virus, would not support replication or mutation of the RCASBP(J)GFP virus.

### Minor points:

1. In abstract remove Only from tumor induction by only two of the variants, since not all variants were tested.

The word was removed (line 32).

2. Please insert a label for x axis (days?) in figure 3 A and B.

The labels were added.

# Part II - Major Issues:

## Reviewer #1:

1. For the eight single nucleotide substitutions discovered, is it possible to conduct further bioinformatics analysis to predict the potential impact of these mutations on viral function and structure?

We sought to predict the effect of individual Env mutations on the interaction with the cell receptor using the current version of AlphaFold3. However, only the prediction of the trimer of the wt SU and TM structure was performed with sufficient reliability. The predicted template modeling (pTM) and interface predicted template modeling (ipTM) scores were as follows:

- SU: ipTM = 0.3, pTM = 0.4
- TM: ipTM = 0.65, pTM = 0.67

Unfortunately, predictions of the interaction of the Env-J trimer with the receptor dimer have very low reliability and are inconsistent with known facts about receptor and Env orientation within the membrane. Therefore, despite our extensive effort, it is not possible to perform any further interaction analysis with current tools.

We conducted bioinformatic analyses of individual substitutions which suggest minor destabilization of most modified Envs, except for the Env with the N311 substitution within the surface unit domain and the A432T substitution within the transmembrane domain. We utilized the tool DDmut (<https://doi.org/10.1093/nar/gkad472>) to predict changes in Gibbs free energy upon point mutations.

| ddG stability (kcal/mol) | |
| --- | --- |
| S236L | -2.38 |
| N311S | -0.91 |
| N311D | -1.34 |
| N311K | 0.1 |
| T313A | -0.27 |
| T313I | -0.49 |
| T313N | -0.16 |
| A432T | 1.34 |

The DDmut tool does not consider glycosylation or interactions with other structures, such as membranes. Additionally, these predictive bioinformatic results contradict our experimental findings, such as the thermostability tests and replication efficiency in DF1 cells. Consequently, we opted not to include these results in the manuscript.

We have added a paragraph concerning this topic into the result and discussion sections as mentioned previously in reaction to reviewer 3 in Part I, point 3.

Results (lines 176 - 178):

S236L, T313A, T313I, and A432T are substitutions that change the hydrophobicity of the side chain, while N311D and N311K are substitutions that change the charge of the side chain.

Discussion (lines 357 - 363):

In contrast, the other two mutated residues are not protruding and probably are not involved directly in the receptor binding. Both S236L and A432T represent substitutions that change the hydrophobicity of the side chain, which could impact the protein folding and influence other steps of virus entry into the cell, such as the membrane fusion process. Mutations can also affect virus stability or protein export efficiency, but given the same viral replication efficiency in the DF-1 cell line, the mutations do not appear to have a significant effect on these properties *in vitro*.

2. Have you considered the potential for these adaptive variants to spread under natural conditions and their impact on poultry populations? In other words, is it currently prevalent for these adaptive mutants within the chicken population?

We mention in the results and in discussion sections that these adaptive mutants were not detected in the chicken populations.

“In available Env of ALV-J (Env-J) sequences (Dataset S1, sheet A) [(Wang et al. 2018)](https://paperpile.com/c/PDkPur/z6VGy), the substituted amino acids are rather invariable. Based on the ratio of non-synonymous to synonymous nucleotide substitutions, the diversifying selection of the substituted amino acid sites was not detected (Dataset S1, sheet B).”

In the Discussion, we consider potential explanations for the absence of these variants in the chicken population. In general, the adaptive variants could spread under natural conditions, but without any clear advantage in chickens that are susceptible to wt ALV-J.

3. The conclusion drawn from result 1 is inappropriate, as the data in this section only counted the number of GFP positive cells, which is insufficient to confirm the claim that a single amino acid mutation confers adaptation of the virus to host resistance. The expression level and viral titer of viral proteins (p27 or/and gp85) should also be tested.
4. Fig2. Replication efficiency of ALV-J variants should be test by other methods, such as viral protein (p27 or/and gp85) expression level of ALV-J, except for the GFP positive cell percentage.
5. In Figure 2, the replication efficiency of the ALV-J variant should be tested using other methods, such as the expression levels of ALV-J viral proteins (p27 or/and gp85), besides the percentage of GFP positive cells.

The common denominator of points 3, 4, and 5 is the requirement to detect viral proteins in addition to GFP detection. Both detection methods have their limitations. The loss of GFP via mutation or recombination may occur during virus replication. Our observations suggest that this loss is more prevalent at lower multiplicity of infection. The loss of GFP can quantitatively shift the characteristics of adaptive mutations, but does not alter the conclusion that a single amino acid mutation confers adaptation of the virus to host resistance. On the other hand, W38-/- cells harbor endogenous retroviruses, whose products can cross react with anti-p27 or anti-gp85 antibodies. We admit that detection of both GFP and viral proteins could be the best option and we therefore performed immunostaining of DF-1 cells infected by RCASBP(J)GFP with high multiplicity. In this case it is apparent that all GFP positive cells are also positive for gag (Fig. 2, letter).

**Fig. 2, letter: Staining of RCASBP(J)GFP infected DF1 cells with Gag antibody and DAPI.**

The blue circle depicts the non-infected cells stained only with DAPI, the red circle depicts Gag positive and GFP-negative cell. Anti-ASLV rabbit p27 Antiserum (Charles River Laboratories, 1:1,000) was used as the Gag antibody.

We have employed the GFP reporter system in previous publications, where it reliably indicated the receptor sensitivity, resistance, and helped to identify critical amino acid residues in receptors [(Koslová et al. 2018; Jiří Plachý et al. 2017; Hughes 2004)](https://paperpile.com/c/PDkPur/354m+GHXX+spRQ). Based on all these facts, we believe that GFP detection is sufficient for conclusions drawn in this manuscript.

6. In Figure 3a, please provide additional details regarding this experimental design in the M&M section. What types of tumors have been induced? In which organs did tumors occur? Images of histopathological changes in the tumor tissue should be provided.

The v-*src* transducing virus induced sarcomas at the side of inoculation in the pectoral muscle as we mention in the section materials and methods. We did not follow the occurrence of distant metastatic tumors in this study and animals have been sacrificed soon after detection of progressively growing sarcomas. We did not perform a rigorous analysis of tumors but a casual histological inspection shows typical structure classified previously as sarcoma/rhabdomyosarcoma. We have added a representative image of the sarcoma *in situ* and a hematoxylin-eosin stained histological section to SI (Fig. S3).

**S3 Fig. Representative sarcoma induced at the site of v-*src* transducing virus inoculation**.

(A) Sarcoma *in situ* in the left pectoral muscle and (B) haematoxylin and eosin staining of a tumor section.

To the chapter ***In vivo* tumor induction in chickens,** we added following details (lines 494 - 496):

The growth of sarcomas at the site of inoculation was monitored (S3 Fig). When the tumor was detected, the bird was euthanized. Tumors were fixed in 4% buffered paraformaldehyde, embedded in paraffin blocks, and processed by routine histological procedures with haematoxylin and eosin staining.

7. To assess the role of detected eight mutations (S236L, N311S, N311D, N311K, T313A, T313I, T313N, and A432T), each of them was only introduced into the RCASBP(J)GFP vector. These results do not fully support this conclusion of single amino acid substitutions within Env-J are sufficient enough to circumvent ΔW38-induced resistance. These eight mutations should be introduced into ALV-J, such as the prototype strain HPRS-103, to further validate their ability to infect W38-/- CEFs.

In this study, we focus exclusively on overcoming ALV-J resistance induced by modification of the cellular receptor. This process occurs at the level of interaction of ALV-J glycoproteins with the respective receptor and can be modeled using RCAS. This system has been used for a long time in our studies on receptors for ALV of different subgroups, leading to the identification of novel receptors or amino acid residues critical for interaction with the virus. Experimentation with real ALV-J, e.g. HPRS103, would be of utmost importance to confirm the model results and to study possible pathogenesis. However, such an experiment would require long-term follow-up (e.g. no one has studied the course of viremia after ALV-J infection) and adherence to safety standards in a dedicated facility.

## Reviewer #2:

1) Given that the obvious goal was to develop resistant chickens, and that the authors are clearly aware that the body temperature of the chickens is significantly higher than 37, which can affect the replication of the mutant viruses (as shown in figure 2), it is not obvious why the selection experiments were done at 37 and not at 42.

We agree with this conceptual comment and are aware of the interpretation difficulties introduced by selection of adaptive mutations at 37°C (point 2). The selection of Env-J mutants followed the methodology employed in previous studies [(Rainey et al. 2003; Munguia and Federspiel 2019; Lounková et al. 2017)](https://paperpile.com/c/PDkPur/xp3x+jOrh+Ti8q). It has often been observed that viruses expanding their host range exhibit increased thermosensitivity compared to wt [(Lounková et al. 2017; Bova-Hill C, Olsen J C, and Swanstrom R 1991; Amberg et al. 2006)](https://paperpile.com/c/PDkPur/Ti8q+91aE+hTWL), hence the cultivation temperature of 37°C is justified.

At the beginning of the project, the adaptive potential of the virus and the number of mutations needed were uncertain. Therefore, the experiments were performed under conditions adjusted for virus replication. Subsequent modifications to these conditions were made based on the observed frequency of adapted virus variants. In the future, it would be interesting to study virus adaptation under conditions better matching the *in vivo* situation.

2) The selections and most of the analysis were done in cultured cells, which is both experimentally simpler and cheaper than in vivo experiments. As shown in figure 3, the in vivo infectivity data in the mutant birds are a better match for the ability of the mutant viruses to grow in tissue culture at 42 than at 37. What is more surprising is that the WT virus produced a tumor in what should have been a stringent in vivo infectivity assay. In that sense, based on an experiment with a small number of birds, the WT virus appeared to be more infectious for the mutant birds in vivo than were several of the mutants that were selected in tissue culture.

As stated in the answer to similar point 4 of the reviewer No. 3, this single tumor induced by a wt virus in W38^-/-^ chicken remains an unexplained exception and we can only speculate that the pseudotyped transforming virus entered the cell by a receptor-independent mechanism. We have added a paragraph in the Discussion, where we point to the observed receptorless virus entry *in vitro* (lines 379 - 386).

Surprisingly, in one instance, a tumor was induced in a W38^-/-^ chicken by a wt variant of the pseudotyped virus. This occurrence was an exception that neither transpired in the previous study nor was replicated in subsequent experiments. Unfortunately, due to the use of pseudotyped viruses, determining the mechanism of tumor induction is not feasible. It could be explained by receptorless cell entry of the transforming virus followed by rescue of v-*src* transducing virus by an endogenous retrovirus of different subgroup specificity. The receptorless entry was observed in our experiments *in vitro* (Fig 4B, Fig S2). When a high multiplicity of infection was used the receptorless RCAS entry was sporadically observed *in vivo* as well [(Elleder et al. 2004; Lounková et al. 2017)](https://paperpile.com/c/PDkPur/0WGc+Ti8q).

In addition, all of the mutant viruses caused tumors in WT birds (although more slowly than WT), which means that the higher temperature in vivo cannot wholly explain the results presented in figure 3. The authors don't comment on either of these unexpected results; they need to discuss the unexpected nature of the data shown in both panels of figure 3.

We are aware that the temperature sensitivity of the adapted virus variants in vitro cannot fully explain the differences in tumor induction in W38-/- and wt chickens. We have added a paragraph in the Discussion in order to provide possible explanations to the results in Fig. 3 (lines 364 - 378).

All adapted variants of the virus tested *in vivo* induce tumors in wt W38^+/+^ birds although more slowly than the wt virus. In W38^-/-^ chickens, however, variants S236L and A432T are inefficient although they are well adapted *in vitro*. We can assume that virus-receptor interactions and subsequent fusion can be affected by a combination of factors, such as sensitivity to higher temperature *in vivo*, decreased virus stability, or sensitivity to lower pH, which may manifest in the acidic tumor environment. According to AlphaFold2 prediction, the substitutions S236L and A432T are not on the top of Env and not exposed for receptor binding. We therefore cannot neglect the possibility that substitutions S236L and A432T destabilize the Env structure and facilitate the release of fusion peptides, so that virus-cell fusion occurs even after suboptimal interaction with the receptor. Such destabilization and partial priming correlate with low thermostability and sensitivity to low pH [(Lounková et al. 2017)](https://paperpile.com/c/PDkPur/Ti8q), which may be a greater barrier in vivo than in vitro. Finally, the Env glycoproteins of adapted variants may present different antigenic epitopes causing slight variance in the immune response [(J. Plachý et al. 1994; Wallny et al. 2006)](https://paperpile.com/c/PDkPur/fQCT+7jXZ). This can be sufficient to prevent tumor growth in W38^-/-^ chicken compared with the situation in wt chickens.

3) The naïve reader may not understand the basis of the in vivo tumorigenesis experiments based on how these experiments are described in the text. The env gene is deleted in the BH-RSV strain. In the in vivo experiments, the RCASBP(J) viruses (WT and mutant) act as a helper virus and supply the env that makes the BH-RSV infectious. Although the resulting BH-RSV is a one round virus, the presence of the RCASBP(J) in the stock means that a cell in a bird that is infected with BH-RSV could be reinfected with the helper virus and, if that happens, BH-RSV can be mobilized in the bird. That could help to explain the difference in the time it takes to see a visible tumor in the experiments shown in figure 3B. Because the helper virus can replicate, there is also the possibility that mutant/revertant viruses could have arisen in some of the in vivo experiments. That possibility suggests that different results might have been obtained if the birds were inoculated either with RCASBP(J) variants that carry src (or some other marker), which would have made it a simple matter to look for env mutants/revertants. Alternatively, if BH-RSV stock had been prepared by transfection with an env expressing plasmid and not a replication competent helper, the BH-RSV virus should not have been able to replicate, or revert/mutate. Whatever the explanation for the data in figure 3B, the nature of the BH-RSV experiments, and the potential implications of the ability of the helper viruses not only to provide an infectious env, but also to replicate, should be discussed.

In order to improve the comprehensibility, we expanded the description of the experiment in the results section (line 220):

Next, we tested the sensitivity of W38^-/-^ chickens to each variant by inducing tumors with a replication-defective v-*src*–transducing virus without env gene, which had been pseudotyped with adapted variants of Env-J.

We have added a paragraph into discussion as we mentioned in our answer to a similar objection in part I, reviewer 3, point 4.

The presence of RCASBP(J) virus means that the BH-RSV can be rescued in the sensitive bird. In W38-/- chicken the tumor induction by wt RCASBP(J) could be explained by receptorless virus entry and/or recombination with some endogenous retrovirus. We admit that the use of RCASBP(J) with *src* marker would be better in future experiments as we discussed previously (Part I, reviewer 3, point 4).

# Part III Minor issues:

## Reviewer #1

1. The scope of the article title is too broad, and only rapid adaptive evolution of ALV-J was detected in the article, without detecting other subgroups, so avian leukosis virus should not be used.

The title was changed.

Rapid adaptive evolution of avian leukosis virus subgroup J in response to biotechnologically induced host resistance

2. Please provide additional details regarding the experimental design in the M&M section, particularly the process of screening and identifying adapted viral variants. For example, why choose to extend the cultivation period by two weeks instead of longer? Will longer cultivation periods produce more adaptive virus mutants?

We have propagated the RCASBP(J)GFP in DF-1 cells for a longer period (up to 79 days). The fraction of the RCASBP(J)GFP virus, which was able to overcome the resistance of W38-/- CEFs was growing steadily (Fig. 3, letter). In our experiment, the RCASBP(J)GFP-infected DF-1 cells were cultured for 21, 43, 59 or 79 days and continuously supplemented with fresh DF-1 cells. After each period, the virus was collected and used for infection of fresh W38-/- CEFs or DF1 cells. The GFP positivity was measured 3 dpi in both cells.

**Fig. 3, letter: Percentage of GFP positive of W38^-/-^ CEF infected with RCASBP(J)GFP collected from DF-1 cell after different cultivation periods.** **The results were normalized to GFP-positive DF-1 cells infected in parallel.**

For the functional analysis of mutations, we selected those that recurred in the early phase of the experiment because they are the best correlate of predicted naturally occurring escape mutations. Later mutations may also be of interest, particularly those that arose in addition to early occurring mutations to compensate for the protein instability imposed by the primary mutations.

## Reviewer #3: Minor points:

1. Please give more detail in text about the reporter virus system used here and the complementation assay.

Details were added into the result section (lines 103-104)

RCASBP(J)GFP is an ALV-based replication competent vector of subgroup specificity J that transduces GFP (Fig. 2A).

2. In abstract remove Only from tumor induction by only two of the variants, since not all variants were tested.

“Only” was removed.

3. Please insert a label for x axis (days?) in figure 3 A and B.

Labels were inserted.

### References:

[Amberg, Sean M., Robert C. Netter, Graham Simmons, and Paul Bates. 2006. “Expanded Tropism and Altered Activation of a Retroviral Glycoprotein Resistant to an Entry Inhibitor Peptide.” *Journal of Virology* 80 (1): 353–59.](http://paperpile.com/b/PDkPur/hTWL)

[Bova-Hill C, Olsen J C, and Swanstrom R. 1991. “Genetic Analysis of the Rous Sarcoma Virus Subgroup D Env Gene: Mammal Tropism Correlates with Temperature Sensitivity of gp85.” *Journal of Virology* 65 (4): 2073–80.](http://paperpile.com/b/PDkPur/91aE)

[Elleder, D., J. Plachý, J. Hejnar, J. Geryk, and J. Svoboda. 2004. “Close Linkage of Genes Encoding Receptors for Subgroups A and C of Avian Sarcoma/leucosis Virus on Chicken Chromosome 28.” *Animal Genetics* 35 (3): 176–81.](http://paperpile.com/b/PDkPur/0WGc)

[Hughes, Stephen H. 2004. “The RCAS Vector System.” *Folia Biologica* 50 (3-4): 107–19.](http://paperpile.com/b/PDkPur/spRQ)

[Koslová, Anna, Dana Kučerová, Markéta Reinišová, Josef Geryk, Pavel Trefil, and Jiří Hejnar. 2018. “Genetic Resistance to Avian Leukosis Viruses Induced by CRISPR/Cas9 Editing of Specific Receptor Genes in Chicken Cells.” *Viruses* 10 (11). https://doi.org/](http://paperpile.com/b/PDkPur/354m)[10.3390/v10110605](http://dx.doi.org/10.3390/v10110605)[.](http://paperpile.com/b/PDkPur/354m)

[Koslová, Anna, Pavel Trefil, Jitka Mucksová, Veronika Krchlíková, Jiří Plachý, Jakub Krijt, Markéta Reinišová, et al. 2021. “Knock-Out of Retrovirus Receptor Gene Tva in the Chicken Confers Resistance to Avian Leukosis Virus Subgroups A and K and Affects Cobalamin (Vitamin B12)-Dependent Level of Methylmalonic Acid.” *Viruses* 13 (12). https://doi.org/](http://paperpile.com/b/PDkPur/YfPV)[10.3390/v13122504](http://dx.doi.org/10.3390/v13122504)[.](http://paperpile.com/b/PDkPur/YfPV)

[Koslová, Anna, Pavel Trefil, Jitka Mucksová, Markéta Reinišová, Jiří Plachý, Jiří Kalina, Dana Kučerová, et al. 2020. “Precise CRISPR/Cas9 Editing of the NHE1 Gene Renders Chickens Resistant to the J Subgroup of Avian Leukosis Virus.” *Proceedings of the National Academy of Sciences of the United States of America* 117 (4): 2108–12.](http://paperpile.com/b/PDkPur/Udo4Z)

[Lounková, Anna, Jan Kosla, David Přikryl, Kryštof Štafl, Dana Kučerová, and Jan Svoboda. 2017. “Retroviral Host Range Extension Is Coupled with Env-Activating Mutations Resulting in Receptor-Independent Entry.” *Proceedings of the National Academy of Sciences of the United States of America* 114 (26): E5148–57.](http://paperpile.com/b/PDkPur/Ti8q)

[Munguia, Audelia, and Mark J. Federspiel. 2019. “Avian Sarcoma and Leukosis Virus Envelope Glycoproteins Evolve to Broaden Receptor Usage Under Pressure from Entry Competitors †.” *Viruses* 11 (6). https://doi.org/](http://paperpile.com/b/PDkPur/jOrh)[10.3390/v11060519](http://dx.doi.org/10.3390/v11060519)[.](http://paperpile.com/b/PDkPur/jOrh)

[Plachý, J., K. Hála, J. Hejnar, J. Geryk, and J. Svoboda. 1994. “Src-Specific Immunity in Inbred Chickens Bearing v-Src DNA- and RSV-Induced Tumors.” *Immunogenetics* 40 (4): 257–65.](http://paperpile.com/b/PDkPur/fQCT)

[Plachý, Jiří, Markéta Reinišová, Dana Kučerová, Filip Šenigl, Volodymyr Stepanets, Tomáš Hron, Kateřina Trejbalová, Daniel Elleder, and Jiří Hejnar. 2017. “Identification of New World Quails Susceptible to Infection with Avian Leukosis Virus Subgroup J.” *Journal of Virology* 91 (3). https://doi.org/](http://paperpile.com/b/PDkPur/GHXX)[10.1128/JVI.02002-16](http://dx.doi.org/10.1128/JVI.02002-16)[.](http://paperpile.com/b/PDkPur/GHXX)

[Rainey, G. Jonah A., Andrew Natonson, Lori F. Maxfield, and John M. Coffin. 2003. “Mechanisms of Avian Retroviral Host Range Extension.” *Journal of Virology* 77 (12): 6709–19.](http://paperpile.com/b/PDkPur/xp3x)

[Wallny, Hans-Joachim, David Avila, Lawrence G. Hunt, Timothy J. Powell, Patricia Riegert, Jan Salomonsen, Karsten Skjødt, et al. 2006. “Peptide Motifs of the Single Dominantly Expressed Class I Molecule Explain the Striking MHC-Determined Response to Rous Sarcoma Virus in Chickens.” *Proceedings of the National Academy of Sciences of the United States of America* 103 (5): 1434–39.](http://paperpile.com/b/PDkPur/7jXZ)

[Wang, Peikun, Lulu Lin, Haijuan Li, Yongli Yang, Teng Huang, and Ping Wei. 2018. “Diversity and Evolution Analysis of Glycoprotein GP85 from Avian Leukosis Virus Subgroup J Isolates from Chickens of Different Genetic Backgrounds during 1989-2016: Coexistence of Five Extremely Different Clusters.” *Archives of Virology* 163 (2): 377–89.](http://paperpile.com/b/PDkPur/z6VGy)

# List of changes:

1. The title was changed.

Rapid adaptive evolution of avian leukosis virus subgroup J in response to biotechnologically induced host resistance

2. “only” was removed (line 32).
3. (line 103 - 104)

RCASBP(J)GFP is an ALV-based replication competent vector of subgroup specificity J that transduces GFP (Fig. 2A).

4. (lines 176 - 178)

S236L, T313A, T313I, and A432T are substitutions that change the hydrophobicity of the side chain, while N311D and N311K are substitutions that change the charge of the side chain.

5. (lines 187 - 196)

…we have performed the interference assay based on competition for receptor and entry interference between viruses of the same subgroup. We preinfected DF-1 cells either with all adapted variants of RCASBP(J)GFP and, two weeks later, we challenged the preinfected cells with wt vector carrying a dsRed fluorescent marker (RCASBP(J)dsRED). We also performed the reciprocal assay with wt RCASBP(J)dsRed as preinfection virus and adapted variants of RCASBP(J)GFP as a challenge. FACS analysis showed no evidence of GFP/dsRED double positive cells in the superinfected DF-1 culture. This strong interference suggests that the adapted variants use the same receptor NHE1 (Fig. 2F). As a control, we used the B subgroup RCASBP(B)GFP vector, which did not interfere with any of the J subgroup viruses.

6. (line 220)

Next, we tested the sensitivity of W38^-/-^ chickens to each variant by inducing tumors with a replication-defective v-*src*–transducing virus without env gene, which had been pseudotyped with adapted variants of Env-J.

7. (lines 357 - 363)

In contrast, the other two mutated residues are not protruding and probably are not involved directly in the receptor binding. Both S236L and A432T represent substitutions that change the hydrophobicity of the side chain, which could impact the protein folding and influence other steps of virus entry into the cell, such as the membrane fusion process. Mutations can also affect virus stability or protein export efficiency, but given the same viral replication efficiency in the DF-1 cell line, the mutations do not appear to have a significant effect on these properties *in vitro*.

8. (lines 364 - 378).

All adapted variants of the virus tested *in vivo* induce tumors in wt W38^+/+^ birds although more slowly than the wt virus. In W38^-/-^ chickens, however, variants S236L and A432T are inefficient although they are well adapted *in vitro*. We can assume that virus-receptor interactions and subsequent fusion can be affected by a combination of factors, such as sensitivity to higher temperature *in vivo*, decreased virus stability, or sensitivity to lower pH, which may manifest in the acidic tumor environment. According to AlphaFold2 prediction, the substitutions S236L and A432T are not on the top of Env and not exposed for receptor binding. We therefore cannot neglect the possibility that substitutions S236L and A432T destabilize the Env structure and facilitate the release of fusion peptides, so that virus-cell fusion occurs even after suboptimal interaction with the receptor. Such destabilization and partial priming correlate with low thermostability and sensitivity to low pH [(Lounková et al. 2017)](https://paperpile.com/c/PDkPur/Ti8q), which may be a greater barrier in vivo than in vitro. Finally, the Env glycoproteins of adapted variants may present different antigenic epitopes causing slight variance in the immune response [(J. Plachý et al. 1994; Wallny et al. 2006)](https://paperpile.com/c/PDkPur/fQCT+7jXZ). This can be sufficient to prevent tumor growth in W38^-/-^ chicken compared with the situation in wt chickens.

9. (lines 379 - 386).

Surprisingly, in one instance, a tumor was induced in a W38^-/-^ chicken by the wt variant of the pseudotyped virus. This occurrence was an exception that did not appear in the previous study nor recurred in subsequent experiments. Unfortunately, due to the use of a pseudotyped virus, the explanation of the tumor induction is only hypothetical. We assume receptorless cell entry of the transforming virus and/or rescue of v-*src* transducing virus by an endogenous retrovirus of different subgroup specificity. Receptorless entry is also indicated by our *in vitro* experiments. (Fig 4B, Fig S2). Using high-multiplicity infection, the receptorless virus entry was sporadically observed in vivo as well [(Elleder et al. 2004; Lounková et al. 2017)](https://paperpile.com/c/PDkPur/0WGc+Ti8q).

10. (lines 494 - 496):

The growth of sarcomas at the site of inoculation was monitored (S3 Fig). When the tumor was detected, the bird was euthanized. Tumors were fixed in 4% buffered paraformaldehyde, embedded in paraffin blocks, and processed by routine histological procedures with haematoxylin and eosin staining.

Figures:

11. Labels for the x axis in Fig 3A and 3B were added.
12. S3 Fig was added.

**S3 Fig. Representative sarcoma induced at the site of v-*src* transducing virus inoculation**.

(A) Sarcoma *in situ* in the left pectoral muscle and (B) haematoxylin and eosin staining of a tumor section.
